# Hepatic abscess in the Spiegel lobe caused by foreign body penetration: report of a case report

**DOI:** 10.1186/s40792-017-0297-z

**Published:** 2017-02-10

**Authors:** Yuki Fujiwara, Hiroaki Shiba, Yukio Nakabayashi, Masahiko Otsuka, Katsuhiko Yanaga

**Affiliations:** 1 Department of Digestive Surgery, Kawaguchi Municipal Medical Center, Saitama, Japan; 2 Department of Surgery, The Jikei University School of Medicine, 3-25-8, Nishi-Shinbashi, Minato-ku, Tokyo, 105-8461 Japan

**Keywords:** Liver abscess, Foreign body penetration, Spiegel lobe

## Abstract

A 69-year-old male patient visited our hospital because of a continuous low-grade fever and right back pain since 2 weeks. Enhanced computed tomography (CT) of his abdomen revealed a mass with ring enhancement (35 × 30 mm) in the Spiegel lobe as well as a needle-like foreign body. Because conservative treatment by fasting and administration of antibiotics was unsuccessful, the patient underwent emergency laparotomy for removal of the foreign body and drainage of the liver abscess. The patient made a satisfactory postoperative recovery without complications and was discharged on the eighth postoperative day. The foreign body was composed of both protein and calcium phosphate and was histologically diagnosed as matured bone. We herein report successful surgical treatment of a patient with a liver abscess in the Spiegel lobe caused by foreign body penetration.

## Background

Unintentional ingestion of a foreign body is not uncommon. Almost all ingested foreign bodies pass through the gastrointestinal tract uneventfully [1]. Obstruction or perforation by ingested foreign bodies are observed in less than 1% of cases, and upper gastrointestinal endoscopy may be helpful for the diagnosis of penetration of the upper gastrointestinal tract by a foreign body [2]. Development of a liver abscess resulting from penetration of a foreign body is rare [3]. We report a case of successful surgical treatment of a liver abscess in the Spiegel lobe caused by foreign body penetration.

## Case presentation

**Fig. 1:**
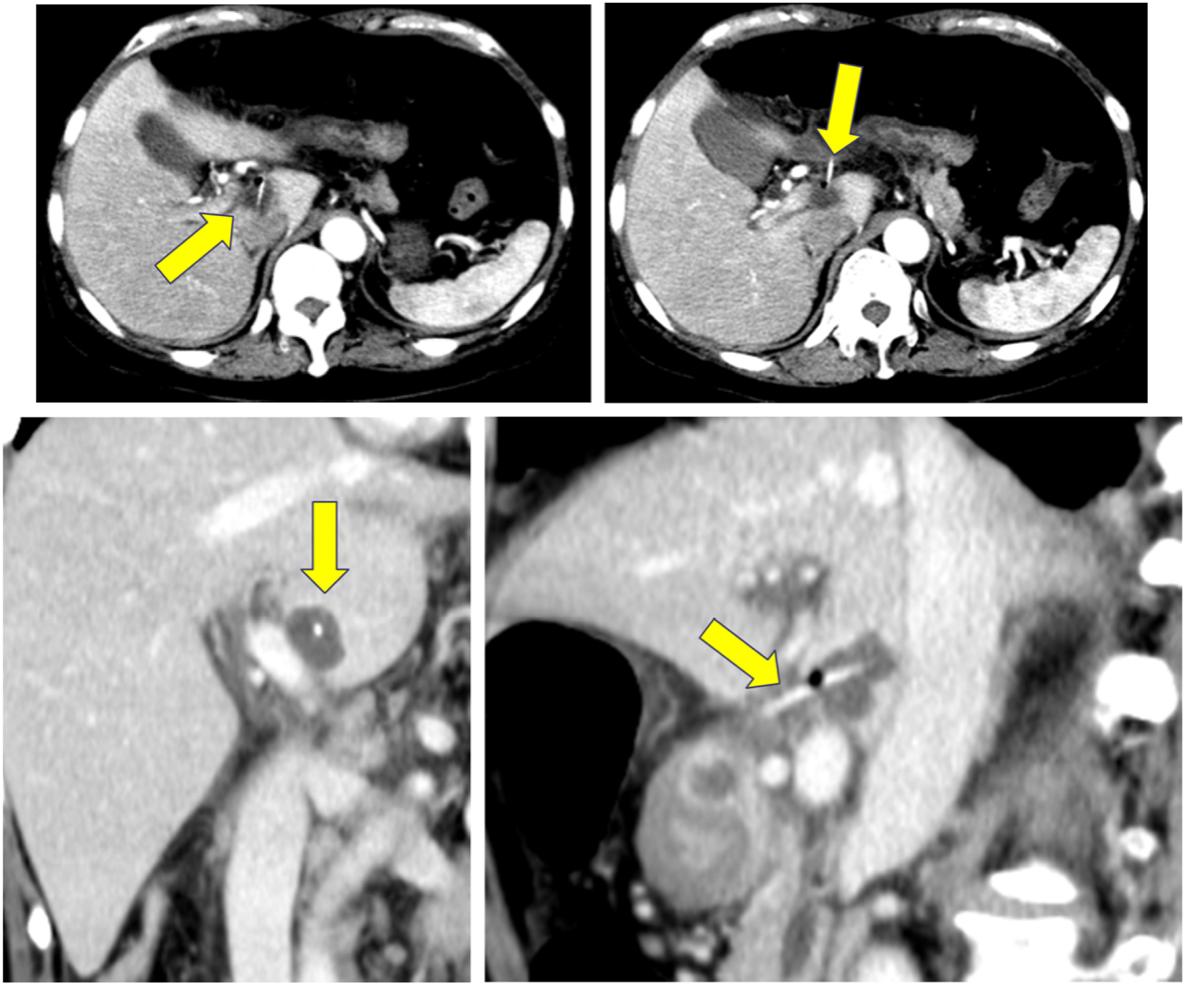
Enhanced computed tomography (CT) revealed a mass with ring enhancement (35 × 30 mm) and a needle-like foreign body. The distance between the tip of the foreign body and inferior vena cava was only 7 mm

A 69-year-old male patient visited our hospital for low-grade fever and right back pain since 2 weeks. Physical examination was unremarkable except for the low-grade fever. Laboratory results showed marked inflammation (C-reactive protein level: 12.94 mg/dl); liver enzyme levels were not elevated. Computed tomography (CT) of the abdomen revealed a mass with ring enhancement (35 × 30 mm) in the Spiegel lobe, which was suspected to be a liver abscess (Fig. 1). A dense, needle-like foreign body that seemed to penetrate the gastrointestinal tract was detected in the abscess. The distance between the tip of the foreign body and inferior vena cava was only 7 mm. The patient had eaten salmon 2 weeks ago. Upper gastrointestinal endoscopy demonstrated no remarkable findings in the stomach or duodenum. Because conservative treatment by fasting and administration of an antibiotic (cefmetazole sodium) was unsuccessful, the patient underwent emergency laparotomy for removal of the foreign body and drainage of the liver abscess. The laparotomy findings showed no ascites or intra-abdominal abscess around the bursa omentalis. However, in the omentulum, there was a liver abscess rolling a foreign body in the Spiegel lobe around the hepatoduodenal ligament.

**Fig. 2:**
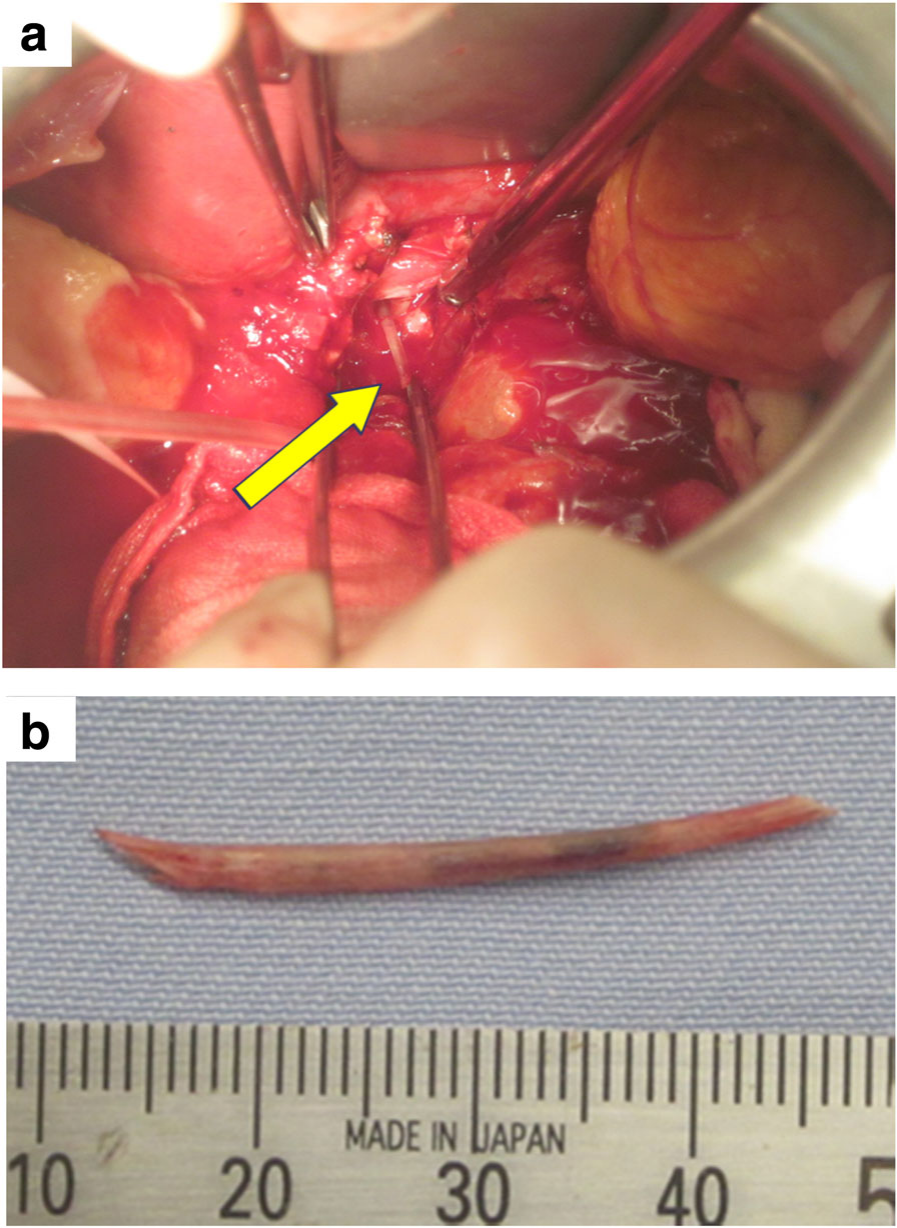
Intraoperative view. The white needle-like foreign body and liver abscess in the Spiegel lobe were found on the left side of the hepatoduodenal ligament (**a**). The needle-like foreign body measured 35 mm in length (**b**)

A white needle-like foreign body was found and removed on the left side of the hepatoduodenal ligament, and the liver abscess in the Spiegel lobe was drained (Fig. 2a). The surgery time was 82 min, and the intraoperative blood loss was 60 ml. The foreign body was composed of protein and calcium phosphate, which was histologically diagnosed as a matured bone (Fig. 2b). Cultures of the abscess fluid grew several types of enterobacterium, including Streptococcus constellatus, Streptococcus milleri, and Streptococcus morbillorum. The patient made a satisfactory recovery without complications and was discharged on the eighth postoperative day.

## Conclusions

The first case of a liver abscess because of gastrointestinal perforation by migration of a foreign body was reported in 1898 [3, 4]. A fishbone is the most frequent cause, accounting for approximately 40% of cases [3]. Chong et al. [5] reviewed the literature for cases of liver abscess secondary to foreign body penetration. They searched the PubMed database for English literature from 1955 to 2013 using the keywords “liver abscess,” “hepatic abscess,” and “foreign body.” Eighty-eight patients were reported in the literature. The left lobe of the liver was the most frequent site of foreign body migration (65.9%) because of the anatomical proximity to the stomach. On the other hand, liver abscesses in the right lobe of the liver mainly occurred by migration of the foreign body (29.5% of cases) from the ascending colon. Bilobar involvement occurred in 4.5% of cases [5]. Liver abscess in the Spiegel lobe is extremely rare, and to the best of our knowledge, this is the first report of liver abscess by foreign body penetration in the Spiegel lobe of the liver. Moreover, in the current case, there were no signs of foreign body penetration, such as purulent ascites or adhesion in the bursa omentalis, from the stomach or duodenum. Generally, when a foreign body penetrates the wall of the gastrointestinal tract, the omentum and other organs seal the perforated gastrointestinal serosa. Therefore, more than half of the cases of viscus perforation required more than 2 weeks to develop symptoms of viscus perforation [6]. In the current case, the mechanism by which the hepatic abscess was caused by a foreign body in the Spiegel lobe of the liver was unclear. We suspect that the foreign body migrated to the Spiegel lobe of the liver through the hepatoduodenal ligament because there were no ascites or intra-abdominal abscess around the bursa omentalis.

One of the most effective treatments for this condition is removal of the foreign body and drainage of the hepatic abscess. Actually, the cure rate without removal of the foreign body is very low [3]. In the review, it was shown that the foreign body was removed by laparotomy or laparoscopic surgery in 54 (61.4%) or 8 (9.1%) patients. As surgical procedures and techniques develop in the future, the number of patients with laparoscopic surgery for removal of the foreign body will increase [5].

The symptoms of liver abscess resulting from foreign body penetration were epigastric pain, low-grade fever, loss of appetite, nausea, and vomiting. Leggieri et al. reported that only 12% of such patients had a suggestive medical history [3]. Enhanced CT is the most important tool for the diagnosis of liver abscess due to a foreign body, which mainly manifests as a calcified linear structure on CT [7]. However, preoperative diagnosis of liver abscesses from a foreign body remains challenging, with the reported incidence of only 25% because foreign bodies are usually small and overlap tissue or fluid [8]. Delayed diagnosis of liver abscess due to a foreign body may lead to poor therapeutic outcome. In the previous report, the mortality rate was 17.6% in 17 cases with liver abscess due to foreign bodies [2].

To the best of our knowledge, this is the first case of successful surgical treatment of a patient with a liver abscess in the Spiegel lobe caused by foreign body penetration.
